# Could a focus on care process put care back into the US health system?

**DOI:** 10.1093/haschl/qxaf022

**Published:** 2025-02-07

**Authors:** Arnav Shah, Reginald (Reggie) D Williams

**Affiliations:** International Health Policy and Practice Innovations, The Commonwealth Fund, New York, NY 10021, United States; International Health Policy and Practice Innovations, The Commonwealth Fund, New York, NY 10021, United States

**Keywords:** national health system comparison, quality of care, care process, prevention, safe care, care coordination, patient engagement

## Abstract

The US health care system continues to underperform compared with other high-income countries, despite excelling on measures of care delivery and quality, also referred to as indicators of “care process.” This commentary explores how the United States managed to perform well on measures of care process and how learning from this lone area of positive cross-country comparison can provide valuable lessons for improving overall health care system performance. By applying these lessons, the United States can create a more effective, efficient, and equitable health care system, ensuring better access, streamlined administration, and improved health outcomes for more Americans.

## Introduction

For 20 years, the Commonwealth Fund has compared health system performance across high-income countries. The most recent edition, “Mirror, Mirror 2024: A Portrait of the Failing U.S. Health System,” ranks 10 high-income countries on 70 measures of health system performance (the 10 countries included in the report are Australia, Canada, France, Germany, the Netherlands, New Zealand, Sweden, Switzerland, the United Kingdom, and the United States). All data in the report are from 2020 or later. Three-quarters of the measures come from the Commonwealth Fund's International Health Policy Surveys from 2021 focused on adults aged 65 years and over, 2022 on primary care physicians, and 2023 on adults aged 18 and over. The “Mirror, Mirror” report is unique in its heavy reliance on survey measures designed to capture the perspectives of patients and professionals—the people who experience health care in each country. In addition to the survey items, standardized data were drawn from recent reports of the Organization for Economic Co-operation and Development (OECD), Our World in Data, the World Health Organization (WHO), publicly and not publicly available country-specific mortality data, the peer-reviewed literature, and from the US Agency for Healthcare Research and Quality.^1^ Authors calculated the overall ranking by averaging together a country's score in 5 key domains: access to care, care process, administrative efficiency, equity, and health outcomes. For years, the United States has spent the most on health care and continued to perform below peer nations. In the most recent report, the United States ranked last overall and came in last or second to last in 4 of the 5 performance domains. The lone exception was care process, which measures care delivery and quality, where the United States ranked second, behind New Zealand and just ahead of the Netherlands. This bright spot of higher performance for the United States offers lessons in how the country could improve access, administrative efficiency, and equity.

The care process domain consists of 36 measures across 4 key topic areas: prevention, safety, coordination, and patient engagement. These areas of focus are meant to capture whether care that is delivered by a country's health care system includes features and attributes that experts consider to be essential to high-quality care. The measures included have proven reliable over several iterations of the “Mirror, Mirror” report.

The United States’ high ranking was driven by above average performance in 3 areas: safety, engagement, and prevention. The United States was a top performer on measures of safe care (ranked first), patient engagement and patient preferences (ranked third), and preventive care (ranked third). The United States scored below the 10-country average on measures of coordinated care, where it came in seventh.

## Factors contributing to US success in care process

In the face of its many well-documented shortcomings, the United States’ relatively high performance on measures of care delivery and quality are worth exploring—where was the United States successful, and what has contributed to the success.

### Patient safety

The United States’ top performance on measures of safety reflects the impact of the American health system's historical focus on patient safety improvements, going back to the late 1990s and the publication of the Institute of Medicine (IOM) report, “To Err Is Human,” which estimated that tens of thousands of patients died from preventable errors in American hospitals each year.^2^

In the 2 decades since the “To Err Is Human” report, US health care leaders have made patient safety a cornerstone of patient care, dedicating extensive research and policy efforts to address it. Organizations like the Institute for Healthcare Improvement and the National Patient Safety Foundation have successfully scaled patient safety interventions.^3^

A study published in the *Journal of the American Medical Association* in 2022 assessed changes in adverse event rates for patient safety between 2010 and 2019. The authors found a significant decrease during that time in the rates of such events for patients with acute myocardial infarction, heart failure, pneumonia, and major surgical procedures. There was also a significant decrease in the adjusted rates of adverse events between 2012 and 2019 for all other conditions.^4^

National policy efforts such as the Patient Safety and Quality Improvement Act of 2005 and the Centers for Medicare and Medicaid Services’ (CMS’) 2011 national initiative, the Partnership for Patients, have worked to meet patient safety goals by supporting local quality-improvement measurement through provision of tools, training, and programmatic structure.^5^ New technologies have emerged in recent years. They can both improve safety and present new hazards.^6^

### Engagement and patient preferences

The engagement and patient preferences domain of the “Mirror, Mirror” report seeks to measure performance on indicators related to having a regular doctor, use of digital or telehealth, how patients are treated when they seek care, how supported by health care professionals patients with chronic illness or near the end of life feel, and levels of primary care physicians’ burnout or emotional distress.

The United States' high performance on measures of telehealth could be attributed to the rapid increase in telehealth availability during the COVID-19 pandemic—public payers like Medicare and Medicaid were allowed to reimburse for certain telehealth services. However, some telehealth regulations tied to the COVID-19 Public Health Emergency expired in 2023, and others are set to expire in 2025. Whether the United States will lead in telehealth availability going forward is still uncertain.

The United States also did well on measures of care for patients with chronic illness, such as discussion of goals and priorities, treatment options, and having a written end-of-life plan. Beginning in 2015, Medicare started reimbursing clinicians for such care-management activities via chronic care management codes.^7^

The United States still faces challenges in areas of engagement, particularly for providers. In the United States, physicians are becoming demoralized by the systemic problems with the health care system that keep them from helping patients.^8^ Physicians across the world have reported high rates of burnout, especially since the COVID-19 pandemic.^9^

### Preventive care

Some of the United States’ relatively higher performance on measures of prevention can be attributed to the Affordable Care Act’s (ACA's) requirement for private insurers to cover more than 100 preventive services without cost-sharing, including vaccinations, screenings for certain chronic conditions and cancers, and preventive medicines.^10^ Research has found increases since the passage of the ACA in rates of blood pressure screenings, cholesterol screenings, colorectal cancer screenings, as well as increased human papillomavirus (HPV) and flu vaccination rates.^10^

The United States had among the highest rates of mammograms for women ages 50–69 years and flu vaccinations for older adults. Breast cancer screening rates in the United States rose steadily in the 1980s and 1990s, thanks to efforts of organizations dedicated to raising awareness of the importance of detecting breast cancer early. Rates in the United States have remained high since the turn of the 21st century, above 70% for the recommended age group, but have largely plateaued due to reduced investments in screening promotion and debates over the optimal age to begin screening.^11^ The Department of Health and Human Services has set a goal of increasing breast cancer screening rates to 80.5% by 2030, which would approach the levels of the leader in the 2024 “Mirror, Mirror” report, Sweden (81%).^12^

Flu vaccination rates for older adults are consistently higher in the United States (71% in 2022) compared to the other 9 countries besides the top-ranked United Kingdom (82%). While much attention has been on vaccination efforts in the wake of the COVID-19 pandemic, in the United States vaccine strategies are more standardized across states than in the European Union.^13^ Efforts to target specific groups, like adults over 65 for influenza, have been more successful in the United States than in other peer countries. However, the United States did rank lower on measles vaccination for children—potentially a reflection of increased skepticism towards certain areas of preventive care among parts of the population.

## Could leaning into care process improve other aspects of system performance?

The US health care system faces unique challenges that even high performance on measures of care process cannot overcome on their own. The United States spends the most, yet has poorer health outcomes. The United States lacks the essential foundation of universal health insurance needed for top performance. Unlike the other 9 countries in the study, a considerable number of Americans remain uninsured—26 million in 2023. Even for those with insurance, Americans face significant affordability barriers. The US system features the largest inequities in treatment—Americans with low incomes often have worse access to care and receive lower quality care. Even with insurance, Americans struggle to determine coverage and affordability, while providers spend significant time navigating insurers. Without improving in these other key areas of performance—access, administrative efficiency, equity, and health outcomes—the US health system will continue to lag behind its peers.

However, care process indicators offer a potential path for improving performance in the other domains measured in “Mirror, Mirror.” The United States performs well on these measures because of a long-term concerted focus on the following: (1) safety, (2) quality improvements, and (3) the use of technology.

Applying the principles and tools that helped the United States perform well on measures of care process can help improve other aspects of performance like access to care, administrative efficiency, equity, and health outcomes. Such actions could include the following:

1. *Using technology to expand access to essential health services*. Implementing evidence-based digital tools and innovations, such as telemedicine and mobile health apps, can significantly enhance the availability and reach of critical health care services, especially in underserved and remote areas.^14^ As clinical medicine transitions from in-person care to digitally enabled care, it is crucial for payment mechanisms to evolve to support new care delivery models.^15^
2. *Leveraging quality-improvement approaches to help drive efficiency and equity practices.* Implementing quality-improvement methodologies like Plan-Do-Study-Act (PDSA) cycle, Root Cause Analysis (RCA), and user-centered design can streamline health care processes, reduce waste, and be used to create accountability in the deployment of equity-focused initiatives.^16^ There is no quality without equity, and there is no equity without quality—quality-improvement efforts should be planned with equity in mind.^17^
3. *Orientating performance measurement and value-based purchasing efforts to addressing leading health outcomes.* Such efforts can drive significant improvements in patient care, which can lead to enhanced quality metrics and cost savings across participating providers.^18^ The CMS Innovation Center has outlined a strategy that shows how this approach could be undertaken in a US context—the Quality Pathway. This initiative aims to align quality goals across payment models, promote the use of person-centered measures of outcomes and experience, and design evaluations to better assess the impact of new models on quality goals.^19^

But is this enough? The United States’ positive care process performance can be replicated in other areas of health system performance—but this will require policymaker action, cross-stakeholder collaboration, and sustained investment in improvement efforts. While the United States continues to lag behind its peers, advancement is possible.

## Supplementary Material

qxaf022_Supplementary_Data
